# Breast Cancer Risk Estimation and Personal Insurance: A Qualitative Study Presenting Perspectives from Canadian Patients and Decision Makers

**DOI:** 10.3389/fgene.2017.00128

**Published:** 2017-09-21

**Authors:** Gratien Dalpé, Ida Ngueng Feze, Shahad Salman, Yann Joly, Julie Hagan, Emmanuelle Lévesque, Véronique Dorval, Jolyane Blouin-Bougie, Nabil Amara, Michel Dorval, Jacques Simard

**Affiliations:** ^1^Department of Human Genetics, Faculty of Medicine, Centre of Genomics and Policy, McGill University Montreal, QC, Canada; ^2^Centre de Recherche du CHU de Quebec, Laval University Quebec, QC, Canada; ^3^Faculty of Science of Administration, Laval University Quebec, QC, Canada; ^4^Faculty of Pharmacy, Laval University Quebec, QC, Canada

**Keywords:** breast cancer, Canada, genetic testing, genetic discrimination, informed consent, insurability, personal insurance, risk

## Abstract

Genetic stratification approaches in personalized medicine may considerably improve our ability to predict breast cancer risk for women at higher risk of developing breast cancer. Notwithstanding these advantages, concerns have been raised about the use of the genetic information derived in these processes, outside of the research and medical health care settings, by third parties such as insurers. Indeed, insurance applicants are asked to consent to insurers accessing their medical information (implicitly including genetic) to verify or determine their insurability level, or eligibility to certain insurance products. This use of genetic information may result in the differential treatment of individuals based on their genetic information, which could lead to higher premium, exclusionary clauses or even the denial of coverage. This phenomenon has been commonly referred to as “Genetic Discrimination” (GD). In the Canadian context, where federal Bill S-201, *An Act to prohibit and prevent genetic discrimination*, has recently been enacted but may be subject to constitutional challenges, information about potential risks to insurability may raise issues in the clinical context. We conducted a survey with women in Quebec who have never been diagnosed with breast cancer to document their perspectives. We complemented the research with data from 14 semi-structured interviews with decision-makers in Quebec to discuss institutional issues raised by the use of genetic information by insurers. Our results provide findings on five main issues: (1) the reluctance to undergo genetic screening test due to insurability concerns, (2) insurers' interest in genetic information, (3) the duty to disclose genetic information to insurers, (4) the disclosure of potential impacts on insurability before genetic testing, and (5) the status of genetic information compared to other health data. Overall, both groups of participants (the women surveyed and the decision-makers interviewed) acknowledged having concerns about GD and reported a need for better communication tools discussing insurability risk. Our conclusions regarding concerns about GD and the need for better communication tools in the clinical setting may be transferable to the broader Canadian context.

## Introduction

Breast cancer is the most common cancer in women worldwide, with nearly 1.7 million new cases diagnosed in 2012 (World Cancer Research Fund)^1^. In Canada, it is estimated that 26 300 women will be diagnosed with breast cancer in 2017 (Canadian Cancer Society)^2^. The risk of breast cancer is determined by a combination of genetic, lifestyle, hormonal and environmental factors (Peto and Mack, 2000). Twin studies suggest familial clustering of breast cancer inherited susceptibility (Lichtenstein et al., 2000; Peto and Mack, 2000). The susceptibility alleles of predisposing genes BRCA1 and BRCA2 are rare in the population and account for 20–25% of the inherited effect on breast cancer (Easton, 1999; Pharoah et al., 2008). The remaining genetic susceptibility to breast cancer is associated with several loci that confer modest independent risk (Pharoah et al., 2002). However, a high proportion of breast cancer occurs in women at a relatively high risk, which highlights the importance of identifying predisposed women in order to facilitate screening and prevention (Peto and Mack, 2000). In this context, common polygenic variants in the general population that are associated with a low cancer-predisposing risk may be more clinically useful for population-screening programs than low-penetrant high-risk predisposition alleles (Pharoah et al., 2008). The distribution of the breast cancer risk based on these common polygenic variations (Michailidou et al., 2013, 2015) is wide enough to allow meaningful distinction between higher and lower-risk groups (Mavaddat et al., 2015). Population screening programs using age as a criterion for eligibility to routine mammography screening might instead exploit individual risk levels based on polygenic variants (Pharoah et al., 2008; Pashayan et al., 2011; Burton et al., 2013; Gagnon et al., 2016).

Beyond expected benefits and hopes from genomic medicine, individuals' risk stratification could also interest third parties outside the medical context, such as insurers. For life insurance underwriting, an individual's insurability and payable premium are established on the basis of one's probability of dying prematurely (Joly et al., 2014, p. 577). For health insurance (ex. long-term care or critical illness), assessing one's insurability can translate into using an individual's genetic test results to predict more accurately the development of future illnesses. Thus, insurers may have an interest in knowing the breast cancer risk estimate or risk stratification level of insurance applicants since it may provide, like other types of predictive medical information, an element to assess insurability and set the corresponding premium amount (CLHIA, 2017, p. 4.6; Joly et al., 2014).

The use of genetic information for underwriting purposes by insurers is associated with an issue referred to as “genetic discrimination” (GD) defined as the differential treatment of a group or an individual based on their genetic data (Otlowski et al., 2012, p. 435). In the context of insurance, GD can take the form of higher premium payments, exclusionary clauses or even denial of coverage. In this light, there has been a legal and policy response in certain regions and countries to prevent GD in personal insurance (Kim et al., 2015). A recent international systematic review on GD in the context of life insurance has shown that there are documented incidents of GD in different countries toward individuals affected by and/or family members at risk of late adult onset monogenic conditions such as Huntington's disease (Joly et al., 2013). However, this study also concluded that outside of these few conditions, there is not enough evidence of systemic discriminatory practices on the part of life insurers (Joly et al., 2013).

Nevertheless, in Europe, human rights provisions have been adopted, in the last 20 years, to provide legal protection against GD in instruments such as the *Convention on Biomedicine* (Council of Europe, 1997), the *Charter of Fundamental Rights of the European Union* (European Union, 2012) and in national legislation of member states having ratified those European norms (Joly et al., 2017). In the United States, the 2008 *Genetic Information Non-discrimination Act* (United States, 2008^3^) protects people from discrimination on the basis of DNA information by health insurers and employers (National Human Genome Research Institute, 2015), while the 2010 *Affordable Care Act* (United States, 2010^4^) prohibits discrimination on the basis of health status by health insurers and explicitly mentions genetic information as a health status related factor (Sarata and Staman, 2015, p. 9; Joly et al., 2017).

In Canada, where most health services are publicly funded, no legislation had been implemented up to recently to provide an explicit protection against GD (Joly et al., 2017, 300). Nevertheless, a federal law, *An Act to prohibit and prevent genetic discrimination* (short title: *Genetic Non-Discrimination Act*)^5^ was recently enacted in order to outlaw discrimination based on genetic test results (Joly et al., 2017, p. 300; Parliament of Canada, 2017). Key provisions of the law provide that individuals cannot be required to undergo genetic testing or to disclose genetic test results (1) in order to have access to goods or services (exception made for research and clinical services) and (2) to obtain or maintain employment (Canada, 3, 6, 8). The law also modifies the Canadian *Human Rights Act* (Canada)^6^ to add “genetic characteristics” as a prohibited ground of discrimination (Canada, 9). However, the *Genetic Non-discrimination Act*^5^ is framed on an old model of genetics (single test for highly penetrant monogenic disorder) that may not capture the full spectrum of GD in this day and age (Joly et al., 2017, p. 301). Its constitutionality may be challenged provinces (House of Commons - Canada, 2016, Bruce Ryder, Peter Hogg; Bouche and Guichon, 2017; Croteau, 2017; Quebec Court of Appeal, 2017). The recently revised *Industry Code: Genetic Testing Information for Insurance Underwriting* of the Canadian Life and Health Insurance Association (CLHIA) states that its members will not require any insurance applicant to undertake genetic testing but that members will require access to the ensuing genetic test results if they were made available to the applicant or her physician (CLHIA, 2017, pp. 4.1–4.2). It also provides that starting January 2018, insurers will not seek results from new genetic tests for applicants holding life insurance policies valued at $250,000 or less (CLHIA, 2017, p. 4.2).

A risk-stratification approach to breast cancer screening and prevention may provide significant health benefits to women. However, the potential impact on the insurability of women at higher risk needs to be fully considered and addressed in the currently changing legal context so they can make informed choices concerning their participation in breast cancer screening programs. Even more so, if such a risk-stratification approach is to be implemented within large-scale public health programs. Health care policymakers may put to good use knowledge about women's perception and attitude on the use of genetic information in insurance for improving communication tools and participation in genetic screening. In this context, it was also important to discuss the issue of insurance and genetics with policy and decision-makers involved in the implementation of these public health screening programs.

Thus, the present study aimed at analyzing perspectives of women as well as decision-makers about genetic testing and insurance as well as general insurability questions in the context of a risk-stratification approach to breast cancer screening and prevention.

## Materials and methods

### Study design

This study is part of a larger research program, PERSPECTIVE, aimed at developing and implementing a personalized risk-stratification approach in order to prevent and detect breast cancer at its earliest stages (Lévesque et al., 2015, p. 283). Considering that this new approach is not yet offered in medical practice and it includes a genetic test component, we focused our study on issues that could be raised by the implementation of a “genetic test” or a “genetic screening test”^7^ to estimate individual risk for screening purpose. This study was approved by the research ethics board of the University Hospital Centre of Quebec.

Our mixed-method study comprised (A) a survey with women and (B) interviews with decision makers:

- The survey with women aimed to capture the knowledge, perspectives, experiences and needs of a sample of Quebec women regarding genetic information and insurance. This survey was administrated on a paper questionnaire at the end of a discussion group centered on the development of an information toolkit for the PERSPECTIVE risk estimation approach. Inclusion criteria for participation in the discussion groups were: being a woman between 35 and 55 years of age, not having received a breast cancer diagnosis, and being able to discuss in French. Each of the four discussion groups were comprised of 8–12 women from different regions of Quebec. Participants were invited to answer our 20-min paper questionnaire consisting of closed-ended questions and the opportunity to comment on their answers. A total of 36 women completed the questionnaire after discussions. This paper discusses only results from the paper questionnaire on insurance concerns (not from discussion groups).
- 14 semi-structured interviews were also conducted with decision-makers involved in the management of breast cancer screening programs and policies in Quebec (Canada). Decision-makers were identified through our network of collaborators and from publicly available sources of information. They were invited to take part in a 1 h-long interview in French, either in person or over the phone, with a researcher trained in sociology and in interview-based research (JH). Our group of decision-makers comprised two clinicians with an active involvement in public health, five experts in public health involved in the evaluation or the implementation of the current breast cancer screening program, four regional managers and three national managers involved in the administration of programs and policies with regards to breast cancer in Quebec (Canada). Interviewees were deliberately selected for maximizing the representation of the following factors: social geography, administrative hierarchy (at the regional and national level), professional experience (clinical, assessment, implementation, and management) and gender balance (Hagan et al., 2016, p. 3). For ensuring a minimum knowledge about the new risk stratification approaches and at the same time foster diversity of opinions among interviewees, half of decision-makers comprised individuals who previously participated in an expert consensus organized by PERSPECTIVE on risk estimation. Interviews were about issues related to risk stratification implementation at the organizational/institutional level (Hagan et al., 2016), including insurability issues raised by genetic testing. Results presented here relate only to parts of these interviews relevant to insurance.

### Data extraction

- The McGill team designed (GD, INF, SS and YJ), collaboratively with the Quebec City team (VD, MD), eight closed-ended questions followed by open-ended questions (blank space to provide an opportunity to explain their answer). The Quebec City Team (VD, MD) hosted discussion groups and distributed the paper questionnaire. Then, the McGill team (GD, INF, SS, and YJ) conducted a content analysis of open-ended answers from participants (qualitative data). Initial results were organized into thematic codes. Responses that could not be categorized were excluded from the analysis. Discrepancies were resolved through majority vote among the researchers. Some participants did not answer each closed-ended and open-ended questions.
- Interviews with decision-makers were conducted (JH) using questions designed by the McGill research team (JH and EL) to guide discussions. The analysis was thematic and the content of each interview was annotated in English. Annotations were discussed among researchers and lead to the development of a themed framework. Triangulation of data, investigators and methodologies was used to derive a more complete understanding of the many dimensions of insurability issues in the context of breast cancer risk stratification assessment (Pope and Mays, 1995).

Due to its qualitative design and the sample size of both the survey participants and interviewees, formal statistical analyses were excluded. Coding of interview content was done in accordance with themes created in survey results.

Survey questions and answers, interview responses and all themes presented in all tables were translated from French by authors.

## Results

### Results of survey with women

#### Demographics of the survey respondents

Respondents provided basic data including their location, age, level of education, occupation/income source and family history of cancer. This information is presented in Table 1. One half of the respondents lived in the two most populous cities of the province of Quebec, namely Montreal (*n* = 6) and Quebec City (*n* = 12). The other half of respondents lives in smaller cities, specifically Rimouski (*n* = 12), Baie-Saint-Paul (*n* = 4), Mont-Joly (*n* = 1), and Saint-Georges (*n* = 1).

**Table 1:** Demographics of survey respondents.

**Demographic Category**	***n***	**%**
Overall	36	
**CITIES WITHIN PROVINCE OF QUEBEC**		
Montreal	6	17
Quebec City	12	33
Rimouski	12	33
Baie-Saint-Paul	4	11
Mont-Joly	1	3
Saint-Georges	1	3
**AGE, YEARS**		
35–40	7	19
41–45	8	22
46–50	7	19
51–56	14	39
Median age = 47.4 ± 6.1		
**EDUCATION**		
University	15	42
Senior high school (CEGEP)	13	36
Secondary	5	14
Primary	1	3
None	2	6
**OCCUPATION/INCOME SOURCE**		
Full-time	17	47
Part-time	5	14
Unemployment benefits	1	3
Social welfare benefits	7	19
Others	5	14
Do not wish to specify	1	3
**FAMILY HISTORY OF BREAST CANCER**		
Yes^*^	17	47
No	19	53

* *Known relatives with cancer (n = 28)*.

The majority of respondents (78%) completed post-secondary education: university degree (*n* = 15) and senior high school diploma (including CEGEP^8^) (*n* = 13). The other respondents only completed secondary (*n* = 5) or primary (*n* = 1) school degrees while a minority did not receive any formal education (*n* = 2).

In terms of occupation/source of income, almost half of the respondents were employed, working full-time (*n* = 17) or part-time (*n* = 5). The second half of respondents received other forms of income including unemployment (*n* = 1), welfare (*n* = 7), invalidity/disability (*n* = 5) or pension payments. We note that one participant did not answer this question (*n* = 1).

Nearly half of the participants shared having a family history of breast cancer (*n* = 17)^9^.

#### Reluctance to undergo breast cancer genetic screening test

Most respondents (*n* = 22) indicated that they would be reluctant to undergo breast cancer genetic screening test knowing results could become accessible to insurers (Table 2). These respondents elaborated on reasons for their choice, which included: being treated differently by insurers was the major concern (*n* = 13), followed by losing current insurance coverage (*n* = 8), being the victim of a data confidentiality breach (*n* = 7), and having to pay increased insurance premiums (*n* = 4).

**Table 2:** Respondents' reluctance to undergo genetic testing.

**Question 1**	**Yes**	**No**
	***n***	***n***
Would you be reluctant to undergo breast cancer genetic testing knowing that results could become accessible to insurers?	22	13
**SUB-THEMES**		
General concern of being treated differently	13	−
Concern about losing insurance	8	−
Concern about the confidentiality of data	7	1
Insurance premium could be increased	4	−
Distrust of insurers	2	−
Genetic testing is for disease prevention only	2	4
Concern that insurers factor cancer family history	1	1
Concern about being treated differently by insurers	1	1
Increased stress	1	−
Diminished freedom of choice in shopping for insurance	1	1
Genetic information is only a risk to develop a medical condition	1	−
Concern that consent is not required for insurers to access applicant's information	−	1
Did not substantiate their position	2	1

A minority of respondents expressed not being reluctant to undergo breast cancer genetic screening test knowing that results could be accessible to insurers (*n* = 13). In this group, some respondents explained their choice by indicating that such tests should be used for the purpose of disease prevention only (*n* = 4).

#### Impact of breast cancer genetic screening test results on insurability

The majority of respondents (*n* = 28) assumed that breast cancer genetic screening test results would negatively impact their ability (or that of a family member) to obtain personal insurance (Table 3). Six of these 28 respondents reiterated this statement in their comments, with many explaining that such test results could represent an additional risk factor for insurance purposes for the person (*n* = 19) or their relatives (*n* = 2).

**Table 3:** Participants' perspectives on insurability impact of breast cancer genetic screening test results.

**Question 2**	**Yes**	**No**
	***n***	***n***
Do you think that breast cancer genetic results would negatively impact your capacity, or that of your relatives, to obtain personal insurance?	28	5
**SUB-THEMES**		
Increased insurability risk for the person	6	−
Genetic test results represent an additional insurance risk factor	19	2
Increased insurability risk for the family	2	−
Diminished freedom of choice in shopping for insurance	1	−
Did not substantiate their position	6	3

On the other hand, a minority of respondents (n = 5) did not think they would experience a negative impact on their ability to obtain insurance. Among them, two respondents nevertheless recognized the risk associated with genetic testing as a general factor used in underwriting by insurers.

When asked whether they had any difficulties in obtaining personal insurance due to their family history of disease or cancer, the majority of respondents (*n* = 29) said “no,” while seven answered positively (Table 4). Six respondents shared knowing someone who experienced difficulties obtaining personal insurance following their genetic test results or that of a relative. These respondents collectively knew 10 individuals who experienced such difficulties with insurance after undergoing a genetic test.

**Table 4:** Respondents' insurance experiences related to family history or results of genetic testing.

**Questions 3 and 4**	**Yes**	**No**
	***n***	***n***
Q3: Did you experience difficulties in obtaining an insurance policy because of your own family history of disease or cancer?	7	29
Q4: Do you know individuals that experienced difficulty in obtaining personal insurance following their own genetic test results or that of a relative?	6	30
Q4b: If yes, how many people?	10 individuals collectively	

#### Insurers' interest in genetic information

A majority of respondents (*n* = 29) thought that insurers have an interest in knowing their genetic information for the purpose of determining their insurability (Table 5). Respondents provided the following explanations about their answer: genetic information is an insurance risk factor (*n* = 12), and it can have a negative impact on insurability (*n* = 5).

**Table 5:** Respondents' perspectives on insurers' interest in genetic information.

**Question 6**	**Yes**	**No**
	***n***	***n***
Do you think that insurers are interested in knowing your genetic information (G.I.) to determine your insurability?	29	5
**SUB-THEMES**		
G.I. is an insurance risk factor	12	−
G.I. has a negative impact in insurability	5	−
G.I. can be a reason for contract nullification by insurers	1	−
Insurers don't use GI for good reasons	1	−
Insurance takers don't trust insurers	1	−
Obtaining personal insurance is easy	−	1
G.I. is not presently an insurance risk factor	1	−
Did not substantiate their position	6	4

*G.I. stands for Genetic Information*.

On the other hand, five respondents did not think that insurers are interested in genetic information.

#### Duty to disclose genetic test results to insurers

The majority of respondents (*n* = 20) thought they did not have a duty to disclose genetic test results in order to obtain a life insurance policy (Table 6). Half of these respondents (*n* = 10) indicated that they lack legal knowledge on this subject, and two respondents stated that genetic information should be treated as an exception to the general requirement of disclosure.

**Table 6:** Respondents' knowledge about the duty to disclose information to insurers.

**Question 7**	**Yes**	**No**
	***n***	***n***
Do you think you have a duty to disclose genetic test results when purchasing personal life policy insurance?	12	20
**SUB-THEMES**		
There is a duty to disclose any health risk to a future insurer	4	0
An insurance contract can be voided if important risks were not disclosed	2	0
General lack of knowledge on the duty to disclose to insurers	1	10
Lack of understanding of the duty to disclose health-related information	1	0
Lack of understanding of insurance contract clauses	1	0
Genetic information should be an exception to the duty to disclose	0	2
Did not substantiate their position	5	8

A minority of respondents (*n* = 12) considered having a duty to disclose genetic test results to prospective life insurers.

#### Disclosure of potential impacts of genetic test results on insurability

Respondents were asked if they should be informed of the potential impact of genetic test results on their capacity to purchase, maintain or renew their insurance policy (see Table 7). Less than half (*n* = 16) of the 36 women answered, but all of these respondents answered yes. Respondents who agreed that women should be informed were invited to answer a sub-question about communication tools. They were asked if information should be included in the “communication tools previously mentioned” or in a “communication tool designed especially” for insurance issues. The “communication tools previously mentioned” could have been understood by women as the website explaining breast cancer risk estimation presented during the group discussions. Some respondents indicated that information on insurability should be included in communication tools previously mentioned (*n* = 7) while others preferred a communication tool designed especially on insurance issues (*n* = 3) (Table 7).

**Table 7:** Respondents' perspectives on the disclosure of potential impacts of genetic test results on insurability.

**Question 8**	**Yes**	**No**
	***n***	***n***
Do you think women should be informed of the potential impacts genetic test results could have on their capacity to obtain, maintain or renew personal insurance?	16	0
SUB-THEMES^*^		
This information should be present in communication tools previously mentioned	7	−
This information should be integrated in specific communication tools	3	−
This information should be integrated in tools easily accessible to women	1	−
There should be a tool to know how to manage and integrate this information	1	−
This information should be provided when a woman requests it	1	−
Insurers should develop a tool to provide this type of information to women	1	−

* Respondents were offered to further explain their answers by answering one of the two following sub-questions: (a) If yes, should this information be integrated in the previously mentioned communication tools or should a more specific tool relating to insurance be more appropriate? (b) If no, why do you think that this kind of information is not necessary?

#### Difference between genetic information and medical information

The majority of respondents (*n* = 22) considered genetic information to be different from other types of medical information (Table 8). Respondents explained their answer by mentioning that genetic information: is personal information (*n* = 11); associated with a risk to develop a future health condition (*n* = 7); and constituted confidential information (*n* = 4).

**Table 8:** Respondents' perspectives on genetic information vs. other types of medical information.

**Question 5**	**Yes**	**No**
	***n***	***n***
In your opinion, is genetic information (G.I.) different from other types of medical information?	22	12
**SUB-THEMES**		
G.I. is personal information	11	1
G.I. relates to a possible future risk	7	1
G.I. is confidential	4	−
G.I. is complex	1	−
There is a distinction between G.I. and health-related data	1	1
G.I. is just one of many types of information	1	4

*G.I. stands for Genetic Information*.

A minority (*n* = 12) of respondents did not consider genetic information to be different from other types of medical data.

### Results of interviews with decision makers

Overall, during the 14 interviews, more detailed discussions occurred on the following themes about insurance (See Table 9): knowledge of the use of medical information by insurers; knowledge about the impact of non-genetic factors on insurability; information and disclosure of a potential impact on insurability; impact on women's participation in genetic screening and; proposed solutions and avenues to explore.

**Table 9:** Themes discussed more in details by decision-makers.

**Decision-makers (*n* = 14)**	**Clinician/public health care (*n* = 2)**	**Regional manager (*n* = 4)**	**National manager (*n* = 3)**	**Expert/public health care (*n* = 5)**	**Total (*n* = 14)**
**THEMES**					
1. Decision-maker awareness on the incidence of GD	2	0	1	2	5
2. Insurers' access to genetic information with the applicant's consent	1	2	1	1	5
3. Genetic vs. other factors in insurance underwriting	2	2	1	3	8
4. Informed consent and the disclosure of insurability risks	2	1	2	1	6
5. Health professional training on disclosure of insurability risks	2	–	–	–	2
6. Impact of informed consent on participation and the fear of GD	2	2	2	4	10
7. Genetic information vs. insurability as a needed ethical, societal debate	–	1	1	1	3
8. The nature of genetic information and its unique character	–	–	1	2	3
9. Potential solutions	–	1	–	3	4

*Numbers indicate how many interviewees addressed the corresponding theme during their interviews. Each decision-maker may have expressed more than one view*.

#### Knowledge of the use of medical information by insurers

When the topic related to the use of medical information (including genetic tests) by insurers was discussed, five interviewees mentioned being familiar with insurers' use of such information in their underwriting processes (Table 9: line 1). Among them, three were uncertain about GD prevalence in the insurance context. One public health expert acknowledged that purchasing certain insurance products might not be a post-test option for some patients who undertake breast cancer genetic predisposition screening, for example if the test reveals a high risk.

Few interviewees (*n* = 5) discussed the interest and means that insurers have to access the genetic information of their applicants (Table 9: line 2). Some of them expressed their knowledge of the current process. One mentioned that insurers expect insurance applicants to disclose to them all relevant health information they are aware of. One stated that insurers generally have a keen interest in accessing medical files and have a lot of resources and expertise to do so. One was unsure if an insurer has the legal capacity to obtain personal medical information directly from the health care system without the consent of the patient, while another interviewee said that an insurer can access the medical information contained in a medical file but only with the patient's consent. One added that once the patient grants access to the medical file, the insurer would surely use this information, including genetic data, to alter premiums or coverage.

#### Knowledge about the impact of non-genetic factors on insurability

A majority of interviewees (Table 9: *n* = 8, line 3) recognized that other factors aside from genetic test results can impact applicants' insurability but are uncertain on their use in underwriting. For instance, two interviewees specified that family history might play a significant role. Moreover, three interviewees thought that insurers could decide to investigate further the possibility of a familial genetic predisposition to a disease based on non-medical data they obtained (e.g., based on patient or family relatives' hospital records). Furthermore, three interviewees recognized their lack of knowledge about the extent of use of both genetic and non-genetic information by insurers for underwriting purposes, which as one interviewee stated, may change depending on different types of insurance products. Likewise, two interviewees were uncertain about the impact of genetic test results on the insurability of asymptomatic women, women who have or maintain a low breast cancer risk, or those having taken preventive actions to reduce a high risk.

#### Information and disclosure of a potential impact on insurability

When discussing informed consent to participate to risk estimation, six interviewees mentioned the impact that genetic test results may have on informed consent and agreed that it should include the disclosure of insurability risks (Table 9: line 4). The importance to inform participants that insurers could have access to this information was mentioned three times, the necessity of disclosing this during pretest genetic counseling was also mentioned three times, and the need to clearly define the scope of the potential impact on insurability was mentioned once. The two interviewees still having an active involvement in clinical practice further added that health professionals should be adequately trained to disclose this potential impact on insurability to women (Table 9: line 5).

Despite some decision-makers being uncertain about the extent of GD in insurance, almost half of interviewees (*n* = 6) believed that women should be informed about the potential impact on insurability before undergoing genetic testing for risk assessment (Table 9: line 4).

#### Impact on women's participation to genetic screening

Without being prompted, many decision makers raised concerns about whether public fears of GD in the insurance context could affect the uptake of breast cancer genetic screening offer. The impact that insurers' access to genetic test results may have on women's participation in said screening was the dominant theme about insurance discussed by a majority of interviewees (Table 9: *n* = 10, line 6). Six of them thought that the anxiety induced by the possibility of being treated differently in the context of personal insurance could undermine women's participation in genetic screening. One regional manager considered that merely rumors of insurers discriminating based on genetics would be enough to lower participation. One public health expert argued that a lack of participation derived from fear of GD in private insurance might impact the rationale behind public genetic screening programs. Only one of the respondents who mentioned the potential impact on insurance considered that the fear of GD would not, alone, be sufficient to affect women's participation in breast cancer screening.

#### Proposed solutions and avenues to explore

Some interviewees (Table 9: *n* = 3, line 7) stated that a public debate should be initiated about the issue of access to genetic information by insurers. One remarked that the confidentiality of patient data is a concern that should be discussed among ethicists. Likewise, one suggested that the use of genetic data from multiple sources (e.g., genetic test results, whole-genome sequencing, and family history of diseases) for underwriting purposes should be reviewed. One recognized the legitimacy of the principle of individual underwriting but would like its application to be constrained in the case of genetic information.

Other interviewees (Table 9: *n* = 3, line 8) discussed the nature of genetic information in the context of insurance underwriting. One thought that the tangible quantitative nature of genetic risk-stratification makes it valuable for insurers when assessing individuals' risk. Two decision-makers reasoned that the use of predictive genetic test results should be limited in underwriting since it conveys only a probability of developing a disease or symptoms.

Some interviewees (Table 9: *n* = 4, line 9) also proposed potential avenues to explore on the issue of insurers' access to genetic test results. One public health expert put forward the idea to limit insurers' access to patients/participants' genetic information in medical records. Another public health expert suggested that access to genetic information by insurers should be prevented. Likewise, two other decision-makers mentioned the potential use of a moratorium on the access and use of genetic information in insurance.

## Discussion

Among themes discussed with women and decision makers, five main themes were chosen for analysis: (1) the reluctance to undergo genetic screening test due to insurability concerns, (2) insurers' interest in genetic information, (3) the duty to disclose genetic information to insurers, (4) disclosure of potential impacts on insurability before genetic testing, and (5) the status of genetic information compared to other health data.

### Reluctance to undergo breast cancer genetic screening test

The majority of our survey respondents (63%, *n* = 22) expressed being reluctant to undergo breast cancer genetic screening test if results were accessible to insurers. Similar concerns have been documented in previous studies involving BRCA1/2 testing such as by Armstrong et al. in 2003 where 55% of the 574 women rated their fear of GD in life insurance as moderate or very important (Armstrong et al., 2003, p. 361). Such concerns may have a negative impact on the uptake of genetic testing or screening offered at large-scale in a public health perspective. For example, in the study conducted by Keogh et al., participants were found to be twice as likely to decline genetic testing after being informed of potential insurance implications (Keogh et al., 2009).

These concerns are not restricted to the context of cancer risk assessment but were also noted toward genetic test results in general. A pediatric study mentioned that 35% of families invited had declined participation in genomic research offering to return research findings to them due to the perceived difficulty of obtaining life and employment insurance in the absence of a specific legal protection against GD in Canada (Stavropoulos et al., 2016). Moreover, the study conducted by Allain et al. showed in a survey of 1699 respondents that 61% were worried about health insurance discrimination when they considered taking a genetic test (Allain et al., 2012, p. 640). In a broader context, 52% of 1513 respondents from a recent large-scale Canadian privacy survey stated being very concerned about undergoing clinically recommended genetic testing if associated results would be available to insurers or employers (Phoenix Strategic Perspectives Inc., 2013, p. 37). Moreover, 71% of those who expressed significant concern respondents (*n* = 1,021) also shared that it would affect their willingness to undergo genetic testing (Phoenix Strategic Perspectives Inc., 2013, p. 38).

An important majority of our survey respondents (78%, *n* = 28) was concerned that breast cancer genetic test results demonstrating a predisposition to develop the disease might have a negative impact on their capability, or that of their relatives, to obtain or maintain insurance. In addition, a majority of survey respondents and decision-makers interviewed in our study felt that genetic test results would be part of factors that insurers take into consideration in the underwriting process. Similarly, a survey from Parkman et al. showed that respondents from United States are either somewhat or very concerned that life insurers might use genetic test results as a factor for determining coverage and costs (Parkman et al., 2014, pp. 516–518). These apprehensions can be considered in light of existing evidence about GD notably in the context of life insurance. For example, a systematic international review on GD (33 studies, which included 14 about breast cancer) identified a small number of GD cases following genetic testing for breast cancer. However, these studies mostly involved breast cancer patients/participants in the United States, Australia and Europe, and data on GD in insurance in Canada remains scarce) (Joly et al., 2013, pp. 5–8).

Since issues related to genetic testing and potential impact on insurability are discussed during pretest genetic counseling (Lane et al., 2015, pp. 1024–1025), the fear of GD causing reluctance to undergo genetic testing can be identified at an early stage, where it may be addressed if researchers or health professionals have the appropriate tools to do so. If informed on this, genetic counselors could also provide information and support to patients regarding their genetic conditions, related potential impacts on insurance and the underlying health decision process. Canadian genetic counselors have previously expressed needing additional information tools on this subject matter for themselves and their patients. (Lane et al., 2015, pp. 1024–1025).

### Insurers' interest in accessing genetic information

The majority of women in our survey (81%, *n* = 29) considered that insurers have an interest in their genetic information for the purpose of assessing their insurability and establishing their premium. Indeed, in the revised *Industry Code on Genetic Testing*, the CLHIA has reiterated that “insurers will not initiate or require any applicant to undergo a genetic test as part of the process of applying for insurance.” (CLHIA, 2017, p. 4.1). Nonetheless, starting January 2018, “for life insurance coverage of more than $250,000, an insurer may request that existing genetic testing results be made available for the purposes of classifying risk.” (CLHIA, 2017, p. 4.2) Insurers also rely on and use information such as family history in their underwriting process. Although the CLHIA's *Industry Code* mentions that “insurers will not require the genetic test results of any person other than the proposed insured” (CLHIA, 2017, p. 4.8), they nonetheless can use the applicant's family history, which could reveal the existence of specific hereditary diseases (CLHIA, 2017, p. 4.9). Moreover, while Canadian insurers are not specifically asking about genetic test results in their insurance proposition forms, questions they use are sufficiently broad and open-ended to trigger such disclosure (Ngueng Feze and Joly, 2014, p. 61). For example, they require information on “tests” completed, recommended or pending without providing any indications as to the specific type of test concerned i.e., whether genetic or not (Ngueng Feze and Joly, 2014, p. 61).

Two decision makers interviewed stated that insurers have a strong interest in accessing genetic information of patients that undertook breast cancer genetic tests. Three stated that insurers have all the expertise and resources necessary to examine other types of information such as family history and hospital records in order to infer a predisposition to breast cancer and that such use could negatively impact a woman's insurability. These perceptions are also present in the survey by Lane et al. which shows comments from Canadian cancer genetic counselors on the insurance practice of inspecting “every part of a patient's health record” in order to find information on disease predispositions (Lane et al., 2015, p. 1026). Like these Canadian cancer genetic counselors (Keogh et al., 2009, p. 1026), some decision makers interviewed (*n* = 3) mentioned being unsure how insurers interpret and use hereditary disease information, genetic and, non-genetic data, in their risk assessment. Two interviewees pointed out that in the case of a screening program aimed at asymptomatic women, the effect of breast cancer genetic test results in underwriting is more problematic because some women—even at low risk—may never develop the disease and yet may still be perceived to be at higher risk than the average population by insurers. Such concerns about insurers' interest in genetic data have been reported in other studies where patients have been reluctant or refused to undergo breast cancer genetic testing or, expressed a preference for paying higher out-of-pocket costs if doing so could potentially prevent insurers from having access to their test results (Armstrong et al., 2003, p. 28; Issa et al., 2013, p. 253).

### The duty to disclose genetic information to insurers

Until recently, Canada had not yet officially adopted specific policies or laws against GD, such that standard insurance law disclosure requirements would apply. For example, art. 2408 of the *Quebec Civil Code* states that an insurance applicant must disclose “all the facts known to him which are likely to materially influence an insurer in the setting of the premium, the appraisal of the risk or the decision to cover it” (Civil Code of Quebec, art. 2408)^10^.

Our study found that most women were not aware of this duty to disclose since more than half of survey respondents (56%, *n* = 20) thought that they were not required to disclose genetic test results to insurers when applying for insurance. Although this has not been explored by our survey, these respondents may also not be aware that insurance contracts usually require that an authorization (i.e., consent) be granted for the insurer to have access to their medical records. This is important since insurers have stated that, with the consent of the applicant, they will seek access to genetic test results that have been made available to the applicant or her physician, as they do for all other types of health-related information^11^ (CLHIA, 2017, p. 4.7).

Studies have found that several health professionals were unaware of or did not fully understand the applicable laws in their jurisdiction, underlining the need for more training or resources for themselves and their patients (Lane et al., 2015, pp. 1025–1026, p. 1031; Pamarti, 2011, pp. 41–42; Huizenga et al., 2010, pp. 253–260).

However, the recently enacted *Genetic Non-Discrimination Act*^5^ creates a criminal prohibition for requiring genetic tests for entering into or continuing a contract or agreement with an individual and sanctions for these prohibited uses of genetic test results across Canada (Canada, 3–7). Since its entry into force (May 4th 2017), standard insurance law disclosure requirements described above would no longer applies to genetic test results. Therefore, despite its *Industry Code*, CLHIA members would no longer be able to require genetic test results for life insurance coverage valued at more than $250 000 (CLHIA, 2017, p. 4.2). Similarly, the CLHIA exemption concerning genetic testing for research purposes would become dispensable (CLHIA, 2017, p. 4.3). Nevertheless, insurers will still be able to ask insurance applicants about their family history, since the prohibition about genetic test results disclosure in the law is only limited to molecular test results (Canada, 2017, p. 2).

Currently, genetic testing for carriers of BRCA 1 and BRCA 2 susceptibility alleles have estimated lifetime breast cancer risk of 60 and 55% that may be of interest for insurance underwriting (Mavaddat et al., 2013). Since the *Genetic Non-Discrimination Act*^3^ prohibits the requirement to undergo genetic testing or disclose genetic test results in order to establish or maintain a contract (while not explicit, this would include insurance contracts), the law seems best adapted to provide protection in the limited context of highly penetrant, familial and well-characterized monogenic diseases that constitute most uncovered instances of GD (Joly et al., 2013, p. 3). Researchers showed that patient risk stratification scores based on common variants with regards to lifetime risk of breast cancer for women range between 8.6 and 24.4% (respectively for the lowest and highest percentile with a first-degree family history of breast cancer; Mavaddat et al., 2015). Therefore, breast cancer stratification scores may be less predictive and also take non-genetic factors into accounts. As such, the law, mostly adapted to single genetic testing, maybe less applicable in the context of the implementation of public screening programs based on polygenic scores.

### Disclosure of potential impacts on insurability

When asked whether potential impacts of genetic test results on insurability should be disclosed to women prior to breast cancer genetic testing, more than half of the survey respondents did not answer this question, which was the last question on the questionnaire. However, all respondents who provided a response (44%, *n* = 16 out of 36) agreed that potential impact on insurability should be disclosed. Decision-makers interviewed in our study agreed that informed consent should include the disclosure of the impact of genetic tests on insurability and that health professionals should be trained to disclose on it. This touches a question underlined by previous researchers about informing patients and research participants about potential impacts of genetic tests results on insurability (where the authors argue that insurability risks related to incidental findings should be an integral part of the consent process and disclosed in consent forms; Apold and Downie, 2011).

There is presently no consensus on the requirement to disclose such risks. Current consent forms used in Quebec for genetic testing are variable, with some including information on potential effect on insurability while others do not (Salman et al., 2016, pp. 38–49). This may create confusion in patients who received little or no information about genetics and insurance and may therefore have a limited awareness of the potential insurability impacts involved. However, an overly alarming wording of the effect on insurability could cause unnecessary anxiety or unwarrantedly deter women from undertaking clinically relevant testing. Indeed, this is a concern shared by decision makers who pointed out that it could be detrimental to the participation to breast cancer genetic screening. This underlines the need to carefully draft and frame the information to be used for insurability risk disclosure during informed consent processes. Moreover, standardized information on the potential impact on insurability should be provided across institutions so that patients giving consent all receive sufficient and accurate information to enable them to make an informed decision. In the development of communication tools on breast cancer risk stratification and insurance, several subjects should be explored including a review and assessment of current legislation pertaining to collection, use, sharing and disclosure of genetic information and their limitations, and review of the data on the incidence of discrimination related to cancer and breast cancer in particular. Harmonization of information on the potential impact on insurability associated with risk assessment needs to take into account its advantages, disadvantages and limits (identified and validated by stakeholders such as interdisciplinary group of physicians, genetic counselors, academics, lawyers and patient representatives in collaboration with the Canadian Association of Genetic Counselors or other comparable independent organizations). The need for additional communication tools and resources for cancer has also been highlighted by genetic counselors and their patients, particularly on the subject of genetic testing and insurability (Lane et al., 2015, pp. 1032–1033). The communication tools could be used and provided by health care professionals involved in stratification assessment consent process such as nurses, general practitioners, genetic counselors and also by health system managers for training and continued education.

As part of PERSPECTIVE, decision-makers were also interviewed in a distinct study on issues pertaining to the influence of organizational factors on the implementation of a breast cancer screening program in the Quebec public health care system (PQDCS) (Hagan et al., 2016). Many interviewed regional managers deemed important to adapt communication strategies to socio-demographic characteristics of local populations (Hagan et al., 2016, p. 6). In this light, regional managers may be better suited to put into place adapted communication tools while national manager may serve to promote harmonized standards. Many decision-makers noted that specialized training in genetics is essential for pre- and post-test counseling, some emphasizing the prominent role that genetic counselors could play in this regard (Hagan et al., 2016, pp. 4–5). Researchers also remarked that while genetic counselors are trained for the evaluation of breast cancer risk, physicians take a more active role in the decision process to determine health management strategies (Amara et al., 2016, p. 1). In the current organizational context, decision makers have to appropriately adapt the implementation of communication tools by improving genetic training with existing staff comprising various health care professionals (e.g., physicians, nurse practitioners or so-called super nurses) and specifying roles in the matter of informing on all risks related to breast cancer screening (Amara et al., 2016, p. 11; Canadian Nurses Association^12^; International Society of Nurses in Genetics; CBC, 2017)^13^.

### Genetic information and other health data

The majority of survey respondents (61%, *n* = 22) consider genetic information to be different from other health data. This finding is not surprising given previous results of respondents' reluctance to undergo genetic testing and their concerns that associated test results may negatively impact their insurability (Phoenix Strategic Perspectives Inc., 2013, p. 37). Thus, the majority of participants could be expected to support the position of genetic exceptionalism, which considers genetic information to be qualitatively and quantitatively different from non-genetic medical information and requiring additional protection (Rothstein, 2005; Adjin-Tettey, 2012, p. 598). Countries following this approach have adopted laws and/or policies to provide a specific protection for genetic data as seen for example in the *Genetic Information Non-discrimination Act*, which applies in the contexts of employment and health insurance underwriting in the United States (United States, 2008^5^).

Two decision makers pointed out that in the context of risk-stratification, where personalized risk is measured in percentages, genetic information is probabilistic, which brings up concerns about how it is being used by insurers in their underwriting process. In view of current concerns about GD, uncertainties related to the nature of genetic information, and its possible impact on participation in genetic screening test, some decision-makers (*n* = 2) have mentioned supporting the adoption of a moratorium on the use of genetic information by insurers while others (*n* = 2) have mentioned that it should be at least regulated. A few decision-makers (*n* = 3) explicitly added that there is a need for a public debate involving ethicists, jurists and law/policy makers on the use of genetic information in insurance. According to them, this debate has to consider: all types of genetic information and not just breast cancer, the confidentiality of genetic data, and whether this information should benefit from an exception in the context of insurance underwriting.

### Limitations of the study

The survey with women included 36 respondents from Quebec, which is a small sample. However, making statistical inference was not planned in our methods, and the composition of the survey group met our goals of diversification of age, occupation, location and education. Another limitation of our study is item nonresponse in the survey, which is significant for some close-ended question such as Q8, the last question on the questionnaire, who has a non-response rate of 56% (*n* = 20). Furthermore, even if most participants answered all closed-ended questions Q1-7, some chose not to share reasons underlying their responses through the optional open-ended format.

The pool of decision makers interviewed was selected through non-probability purposive sampling, a method aimed at maximum variation rather than statistical generalizations. While within sample variation was attained with regards to geographical location, professional expertise, administrative hierarchy and gender, two limitations remain. The first one lies within the intentional over-representation of decision-makers knowledgeable about the new breast cancer risk-stratification approach. The second one is the potential for interviewer bias. Since many interviewees were not familiar with insurability issues, the interviewer had to rely on informal prompts, which increase the possibility of influencing interviewees' answers.

Since the conduct of both the survey and interviews, Bill S-201 has been enacted by the Parliament of Canada (Parliament of Canada, 2017). While our discussion and analysis take into account this recent legislative development, it is important to note that participants in both survey and interviews may have limited knowledge of it. It is also worth noting that despite some policy and legal duties that may apply specifically to the province of Quebec, our conclusions regarding concerns about GD and the need for better communication tools discussing insurability risk in the clinical setting may be transferable to the broader Canadian context.

## Conclusion

Findings from this mixed-method study show that an important proportion of responding women from the general public who never had a breast cancer diagnosis express being reluctant to undertake genetic testing for breast cancer due to insurability concerns. Their reasons seem to be based on the assumption that genetic test results may have a negative impact on their insurability. The majority of women surveyed consider that insurers have an interest in knowing their genetic information for the purpose of insurance underwriting. Thus, upon being informed of the potential of genetic test results being accessible to insurers, women may be reluctant to undertake breast cancer genetic screening test or assessment. Furthermore, our study results also show that decision makers interviewed have concerns that women apprehension of GD in the insurance context might impede participation in genetic screening programs. These concerns appear to be enhanced by the uncertainty surrounding the use of genetic data that insurers may access.

Despite the fact that published data on the incidence of GD are rare outside of late adult onset monogenic conditions, such as Huntington's disease, the general concern about GD may be a barrier to the uptake of genetic screening test or its implementation. In light of the recent entry into force of Bill S-201, possible constitutional challenges and the recent revisions made to the CLHIA *Insurance Code*, there is a greater need for innovative collaborative communication strategies about the impact of these developments to inform people at risk of being discriminated. Communication strategies should aim to foster a thorough understanding of the insurance applicant's duties, if any, and provide a clear picture of conditions for insurers' use of genetic information through the development of legally accurate, accessible and nuanced information tools collaboratively designed by policymakers, patient organizations and other key stakeholders.

## Ethics statement

This study was carried out in accordance with the recommendations of the research ethics board of the University Hospital Centre of Quebec with written informed consent from all subjects. All subjects gave written informed consent in accordance with the Declaration of Helsinki. The protocol was approved by the research ethics board of the University Hospital Centre of Quebec.

## Author contributions

The Mcgill team GD, IN, SS, and YJ designed, collaboratively with the Quebec City team VD and MD, all survey questions. The Quebec City Team VD and MD hosted the discussion groups and distributed the paper questionnaire. Interviews with decision-makers were conducted JH using semi-structure questionnaire designed by the McGill research team JH and EL to guide the discussions. The McGill team GD, IN, SS, and YJ conducted a content analysis of data collected from participants (qualitative data). All authors made a substantial contribution to the analysis and interpretation of data, provided a critical revision and agreed with the final version of the manuscript and all aspects of the work.

### Conflict of interest statement

The authors declare that the research was conducted in the absence of any commercial or financial relationships that could be construed as a potential conflict of interest.

## Footnotes

^1^World Cancer Research Fund Breast Cancer Statistics. World Cancer Research Fund International. Available online at: http://www.wcrf.org/int/cancer-facts-figures/data-specific-cancers/breast-cancer-statistics (Accessed April 25, 2017).

^2^Canadian Cancer Society Breast cancer statistics. www.cancer.ca. Available online at: http://www.cancer.ca/en/cancer-information/cancer-type/breast/statistics/?region=bc (Accessed July 10, 2017).

^3^United States (2008). Genetic Information Nondiscrimination Act of 2008. Available at: https://www.eeoc.gov/laws/statutes/gina.cfm (Accessed August 23, 2013).

^4^United States (2010). Affordable Care Act. Available at: https://www.gpo.gov/fdsys/pkg/PLAW-111publ148/pdf/PLAW-111publ148.pdf (Accessed April 26, 2017).

^5^Canada Genetic Non-Discrimination Act. Available online at: https://www.canlii.org/en/ca/laws/astat/sc-2017-c-3/latest/sc-2017-c-3.html (Accessed May 30, 2017).

^6^Canada Canadian Human Rights Act. Available online at: https://www.canlii.org/en/ca/laws/stat/rsc-1985-c-h-6/latest/rsc-1985-c-h-6.html (Accessed October 27, 2015).

^7^Better understood by women and decision makers than the “risk stratification” concept.

^8^It refers to the public post-secondary education collegiate institutions exclusive to the education system in the province of Quebec (Canada).

^9^For the purpose of this question, the term “family member” was defined to include biologically related individuals: father, mother, children, brothers and sisters, half-brothers and half-sisters, aunts and uncles, nieces and nephews, cousins and grandparents. For the purpose of determining hereditary breast cancer history, non-biological kin such as parents-in-law, brothers and sisters-in-law, adoptive parents and their children, children of new partners (whether married or not with the participant) and adopted children were not considered family members.

^10^Civil Code of Quebec. Available at: https://www.canlii.org/en/qc/laws/stat/lrq-c-c-1991/latest/lrq-c-c-1991.html#sec1370 (Accessed June 18, 2014).

^11^Starting January 2018, there will be an exception made for applicants seeking life insurance policies valued below $250,000 (CLHIA, 2017, 4.2).

^12^Canadian Nurses Association Nurse Practitioners Available online at: https://cna-aiic.ca:443/en/professional-development/advanced-nursing-practice/nurse-practitioners (Accessed July 10, 2017).

^13^International Society of Nurses in Genetics Home. Available online at: http://www.isong.org/ISONG_genetic_nurse.php (Accessed July 10, 2017).
